# Influence of recombinant S2 cell population enrichment on rabies virus glycoprotein expression and especific RNA and DNA quantities

**DOI:** 10.1186/1753-6561-8-S4-P230

**Published:** 2014-10-01

**Authors:** Nayara Santos, Carlos Pereira, Daniella Ventini-Monteiro, Ana Lia Puglia, Renato Astray

**Affiliations:** 1 Instituto Butantan, São Paulo, SP, Brazil

## Background

Different strategies have been evaluated for increasing the productivity of *Drosophila melonogaster *(S2) cells expressing recombinant rabies virus glycoprotein (RVGP). The maintenance of selective pressure during all the cultivation time and new methods for population enrichment with high level expressing cells are two methods which can increase the productivity through increased expression of RVGP gene. To understand how these methods can improve glycoprotein expression, analysis of the quantities of heterologous DNA and RNA were performed [1,2].

## Methods

From S2MtRVGPHy cell population (untreated control) three populations were generated: S2MtRVGPHy+Hy was obtained after selective pressure using hygromycin for 2 weeks; S2MtRVGPHy-M2 and S2MtRVGPHy-M3 were obtained after immunomagnetic enrichment of RVGP expressing cells (MACS, Miltenyl Biotec), using rabbit polyclonal antibody and mouse monoclonal antibody, respectively [3]. Cell populations were induced with CuSO_4 _for RVGP expression and submitted to cycloheximide (CHX) treatment before sampling in different periods. Samples were analyzed by ELISA, qPCR and qRT-PCR.

## Results

All cell populations presented no significative differences in RVGPDNA content as analysed by qPCR. Before treating the cultures with CHX, cell populations showed very similar amounts of RVGPmRNA and were expressing RVGP at concentrations of 23.1, 31.9, 65.3 and 45.7 ng / 10^6 ^cells for S2MtRVGPHy, S2MtRVGPHy-M2, S2MtRVGPHy-M3 and S2MtRVGPHy+Hy, respectively, showing that both population enrichment strategies were successful on improving RVGP expression. When CHX was added to cultures, the amounts of RVGP decreased probably due to degradation and translation interruption [4]. As expected, RVGPmRNA levels increased.

## Conclusion

Immunomagnetic enrichment of RVGP expressing cells and hygromycin treatment showed to be efficient on increasing RVGP productivity. These methods are easy to perform and may be used for additional selection of recombinant S2 cell populations that are unable to clonal selection because of absence of growth in low cell concentrations. As these methods not statistically changed the amount of RVGPDNA copies / cell between the cell populations in study, the differences in RVGP expression could be attributed to different transcription and translation rates. For studying expression profiles, an inhibitor of translation in eukaryotes (CHX) was added to cultures for translation blockage. The amounts of accumulated RVGPmRNA showed that cell populations exhibited different profiles of transcription and translation for glycoprotein expression. While S2MtRVGPHy-M3 produced the highest level of RVGP (65.3 ng/10^6 ^cells), it showed the smallest level of RVGPmRNA accumulation (R = 5.7) among all populations. As S2MtRVGPHy+Hy produced the second highest level of RVGP (45.7 ng/10^6 ^cells), and showed the highest RVGPmRNA accumulation level (R = 499.4), probably cells present different rates of processing due mainly to metabolic differences. Future experiments undergoing more broadly kinetic evaluations of RVGP and RVGPmRNA may contribute do better understand these results.
